# Unveiling the predictive synergy of red cell distribution width and peak morning home systolic blood pressure: a dual biomarker approach for cognitive risk stratification in hypertensive minor stroke

**DOI:** 10.3389/fneur.2025.1637561

**Published:** 2025-08-26

**Authors:** Yimin Shu, Jie Chen, Yanjin Song, Wuhong Wang

**Affiliations:** Department of General Practice, People's Hospital Affiliated to Ningbo University, Ningbo, China

**Keywords:** red cell distribution width, peak morning home systolic blood pressure, hypertension, cognitive impairment, minor stroke

## Abstract

**Aim:**

This study aims to evaluate the predictive value of combining red cell distribution width (RDW) and peak morning home systolic blood pressure (SBP) for post-stroke cognitive impairment (PSCI) in hypertensive patients with minor stroke.

**Methods:**

A prospective cohort study enrolled 430 patients, randomly stratified into training (*n* = 301) and validation (*n* = 129) cohorts. Multivariable logistic regression and random forest model were employed for analysis.

**Results:**

Both RDW and peak morning home SBP emerged as independent risk factors for PSCI (OR = 70.95 and 1.104, respectively; *p* < 0.001 for both). The combined model significantly improved predictive performance, achieving an area under the curve (AUC) of 0.925 (95% CI: 0.901–0.948), surpassing individual biomarkers (RDW: AUC = 0.883; peak morning home SBP: AUC = 0.765). Subgroup analyses demonstrated superior discriminative capacity in coronary heart disease (AUC = 0.956) and female patients (AUC = 0.925).

**Conclusion:**

The integration of RDW and peak morning home SBP provides an efficient, cost-effective, and clinically translatable tool for cognitive risk stratification in hypertensive patients with minor stroke, offering enhanced precision over conventional single-biomarker approaches.

## 1 Introduction

Stroke remains a leading global cause of mortality and long-term disability, with its burden escalating alongside aging populations. Recent estimates indicate that stroke-related costs exceed $890 billion worldwide, disproportionately affecting low-income countries where 87% of stroke-associated deaths occur, primarily linked to metabolic, environmental, and behavioral risk factors (1). Among stroke subtypes, minor stroke, characterized by low National Institutes of Health Stroke Scale (NIHSS) scores and preserved neurological function, constitutes a substantial proportion of cases. Despite mild initial symptoms, these patients frequently develop significant cognitive dysfunction (2, 3), motor impairments [e.g., dysphagia (4), visuoemotional recognition deficits (5)], and even psychiatric sequelae such as depression (6), collectively undermining quality of life and functional independence (7, 8).

Post-stroke mild cognitive impairment (MCI), a prevalent complication, not only heralds dementia progression (9, 10) but also elevates recurrent stroke risk. Early identification of patients at high risk for cognitive deterioration is thus clinically imperative. However, existing tools for predicting post-stroke cognitive impairment (PSCI) suffer from limited accuracy and scalability, necessitating novel, cost-effective, and non-invasive biomarkers for clinical risk stratification and targeted interventions.

Red cell distribution width (RDW), a routine hematologic parameter quantifying heterogeneity of erythrocyte volume, has recently emerged as a surrogate marker of systemic inflammation, oxidative stress, and endothelial dysfunction (11–13). Multiple studies have confirmed that elevated RDW independently predicts adverse cardiovascular outcomes, including stroke and cognitive decline (14, 15). While its prognostic role in stroke is increasingly recognized, evidence linking RDW to PSCI, particularly in minor stroke populations, remains sparse.

Concurrently, blood pressure variability, especially home-measured values, has gained attention as a modulator of cognitive trajectories in recent years (16, 17). It has been shown that home blood pressure can serve as a traditional and daily predictor of cognition in older adults (18). Another study has revealed that home blood pressure, surpassing office blood pressure, demonstrates higher diagnostic efficiency for MCI in hypertensive individuals (19). Notably, peak home blood pressure has emerged as a critical predictive indicator (20). Home blood pressure monitoring (HBPM), owing to its superior reproducibility and ability to reflect true physiological states, has gradually replaced traditional office blood pressure measurement, becoming an ideal tool for detecting blood pressure fluctuations (21, 22). The present study focuses on the predictive role of peak morning home systolic blood pressure (SBP) in cognitive dysfunction.

This study investigates the joint predictive capacity of RDW and peak morning home SBP for cognitive impairment in hypertensive patients with minor stroke. We hypothesize that both biomarkers independently predict cognitive decline and exhibit superior discriminative performance when combined. Cognitive function was assessed at 3 months post-stroke using the Montreal Cognitive Assessment (MoCA). Validation of this hypothesis could advance precision medicine by providing a new dual biomarker strategy for early PSCI detection and personalized intervention.

## 2 Materials and methods

### 2.1 Study design

A prospective cohort study was conducted in which hypertensive patients with minor stroke were consecutively recruited at our hospital from January 2019 to September 2024. Each enrolled participant was followed for 3 months after admission to monitor the occurrence of post-stroke cognitive impairment (PSCI). The final follow-up visit was completed in December 2024. Based on the accumulated data, a risk prediction model for PSCI was subsequently developed. Inclusion criteria comprised: 1. NIHSS score ≤ 3; 2. presentation with minor stroke symptoms at admission (MoCA score ≥ 26), supported by magnetic resonance imaging (MRI) and/or computed tomography (CT)/CT angiography (CTA); 3. age ≥ 18 years; 4. intact consciousness without comprehension deficits, and no history of major psychiatric disorders, alcohol dependence, or substance abuse. Exclusion criteria included: 1. pre-existing/suspected cognitive impairment, or dementia attributable to other etiologies; 2. prior thrombolysis or endovascular therapy; 3. comorbid severe organic diseases such as myocardial infarction, hepatic/renal/pulmonary insufficiency, or malignancy. A total of 430 eligible patients were ultimately included in the final analysis. The study protocol was approved by the Ethics Committee of People’s Hospital Affiliated to Ningbo University Hospital, and written informed consent was obtained from all participants prior to enrollment.

### 2.2 Cohort stratification and covariate assessment

Patients were stratified into training (*n* = 301) and validation (*n* = 129) cohorts at a 7: 3 ratio using the R function “createDataPartition” to ensure balanced outcome event distribution between groups. The training cohort served for variable selection and model development, while the validation cohort was reserved for external performance evaluation. Guided by established stroke risk frameworks, we systematically collected covariates with potential confounding effects on stroke outcomes. Demographic characteristics, including age, gender, height, body mass index (BMI), residential setting, marital status, and educational attainment, were obtained through standardized face-to-face interviews conducted in the hospital during the admission period. Clinical parameters were extracted from the electronic medical record system of our hospital, encompassing onset-to-admission time, length of hospital stay, admission NIHSS score, MoCA score at admission, admission diastolic blood pressure (DBP)/SBP, risk factors [diabetes mellitus, hyperlipidemia, coronary heart disease (CHD), pre-existing or newly diagnosed atrial fibrillation, transient ischemic attack (TIA) history, prior stroke], and stroke etiology classification (small vessel disease, large artery occlusion, cardioembolism, other determined causes, undetermined etiology). Laboratory biomarkers analyzed within 1 day after admission included serum glycohemoglobin (HbA1c), total cholesterol (TC), triglycerides (TG), high-density lipoprotein cholesterol (HDL-C), low-density lipoprotein cholesterol (LDL-C), uric acid (UA), and interleukin-6 (IL-6).

In total, 430 participants were included in this study. The age of participants ranged from 45.9 to 82.1 years. The duration of education ranged from 0 to 20 years, with a median of 3 years. The cohort included 216 males and 214 females. Body mass index (BMI) ranged from 21.0 to 28.0 kg/m^2^, and admission NIHSS scores ranged from 1 to 3. Detailed distributions of other demographic and clinical characteristics are provided in Table 1.

**Table 1:** Baseline data comparison [x̄ ± s, *n* (%), M (P_25_, P_75_)].

Variables		Non-CD Group (*n* = 258)	CD Group (*n* = 172)	*t*/χ2/*Z*	*p*
Age (years)		62.92 ± 6.10	63.18 ± 4.91	0.467	0.641
Gender	Male	140 (54.26%)	74 (43.02%)	5.216	0.022
	Female	118 (45.74%)	98 (56.98%)		
Height (cm)		154.72 (160.27, 165.07)	159.57 (155.02, 164.94)	−0.055	0.956
BMI (kg/m^2^)		24.47 (22.70, 26.15)	24.27 (22.48, 26.10)	−0.183	0.855
Onset-to-admission time (days)		8.00 (6.00, 12.00)	9.00 (7.00, 11.75)	−0.897	0.370
Length of hospital stay (days)		15 (10.00, 21.00)	17.00 (11.00, 22.00)	−1.221	0.222
Admission NIHSS score		2.00 (1.00, 3.00)	2.00 (1.00, 3.00)	−0.471	0.638
Educational attainment (years)		4.00 (1.00, 6.00)	3.00 (1.00, 4.00)	−4.270	<0.001
Admission SBP (mmHg)		160.52 (150.17, 169.75)	158.51 (150.01, 170.38)	−0.490	0.624
Admission DBP (mmHg)		96.56 (90.38, 103.40)	96.32 (89.83, 103.87)	−0.061	0.951
Residential setting				0.929	0.629
Urban		93.00 (36.05%)	61.00 (35.47%)		
Rural		78.00 (30.23%)	46.00 (26.74%)		
Suburban		87.00 (33.72%)	65.00 (37.79%)		
Marital status				3.459	0.177
Unmarried		34.00 (13.18%)	14.00 (8.14%)		
Married		179.00 (69.38%)	132.00 (76.74%)		
Others		45.00 (17.44%)	26.00 (15.12%)		
Risk factors					
Diabetes mellitus		73.00 (28.29%)	46.00 (26.74%)	0.124	0.725
Hyperlipidemia		100.00 (63.29%)	61.00 (84.72%)	0.478	0.489
CHD		94.00 (36.43%)	41.00 (23.84%)	7.603	0.006
Pre-existing atrial fibrillation		55.00 (21.32%)	33.00 (19.19%)	0.288	0.591
Newly diagnosed atrial fibrillation		37.00 (14.34%)	21.00 (12.21%)	0.402	0.526
TIA history		63.00 (24.42%)	44.00 (25.58%)	0.075	0.785
Prior stroke		110.00 (42.64%)	81.00 (47.09%)	0.831	0.362
Etiology classification				3.430	0.489
Small vessel disease		35.00 (13.57%)	17.00 (9.88%)		
Large artery occlusion		41.00 (15.89%)	22.00 (12.79%)		
Cardioembolism		30.00 (11.63%)	24.00 (13.95%)		
Other determined causes		67.00 (25.97%)	42.00 (24.42%)		
Undetermined etiology		85.00 (32.95%)	67.00 (38.95%)		
HbA1c%		5.93 (3.92, 7.79)	6.30 (3.52, 8.96)	−1.498	0.134
TC (mmol/L)		4.03 (3.40, 4.52)	3.99 (3.42, 4.44)	−0.616	0.538
TG (mmol/L)		1.84 (1.19, 2.41)	1.81 (1.33, 2.53)	−0.966	0.334
HDL-C (mmol/L)		1.43 (0.75, 2.26)	1.80 (0.98, 2.49)	−2.079	0.038
LDL-C (mmol/L)		2.07 (1.79, 2.38)	2.06 (1.80, 2.39)	−0.333	0.739
UA (μmol/L)		345.19 (342.03, 349.07)	345.32 (341.86, 349.06)	−0.110	0.912
IL-6 (pg/ml)		8.70 (4.05, 12.25)	8.80 (4.68, 12.26)	−0.930	0.352
RDW (%)		13.33 (12.80, 14.17)	14.22 (14.00, 14.47)	−13.449	<0.001
Average peak morning home SBP (mmHg)		155.48 (143.62, 165.28)	167.47 (158.54, 174.34)	−9.322	<0.001
Average peak morning home DBP (mmHg)		97.14 (91.20, 102.73)	96.94 (90.61, 102.64)	−0.311	0.756

CD, cognitive decline; BMI, body mass index; NIHSS, National Institutes of Health Stroke Scale; SBP, systolic blood pressure; DBP, diastolic blood pressure; CHD, coronary heart disease; HDL-C, high-density lipoprotein cholesterol; LDL-C, low-density lipoprotein cholesterol; TIA, transient ischemic attack; HbA1c, glycohemoglobin; TC, total cholesterol; TG, triglycerides; UA, uric acid; IL-6, interleukin-6; RDW, red cell distribution width.

### 2.3 Measurement of RDW

RDW quantifies erythrocyte volume heterogeneity, serving as a clinical indicator of systemic inflammatory status and anemia classification. Venous blood samples were collected from all enrolled patients within 24 h post-admission under fasting conditions using heparinized vacutainers. Following collection, samples were immediately subjected to gentle inversions to ensure homogeneous anticoagulant distribution while preventing erythrocyte aggregation. RDW analysis was performed on a fully automated 5-part differential hematology analyzer (Jiangxi Tecom Biotechnology Co., Ltd., Jiangxi Medical Device Registration Certificate No. 20172220254) following standardized operational protocols.

### 2.4 Measurement of peak morning home SBP

Home blood pressure monitoring (HBPM) was performed following international recommendations (23, 24). All participants measured their blood pressure at home using a validated automatic oscillometric device (YE660D, Yuwell Medical Equipment Co., Ltd., China). Measurements were conducted each morning for 7 consecutive days, within 1 h after awakening, after voiding, before breakfast, and prior to taking antihypertensive medication. During each session, participants took three consecutive readings at 15-s intervals while seated and after resting quietly for at least 5 min, with the arm supported at heart level. All readings were stored in the device’s memory with automated time stamps. Data were reviewed and extracted at outpatient follow-up visits by a blinded research assistant to ensure protocol adherence.

The average peak morning home systolic blood pressure (SBP) was defined as the mean of the three highest morning SBP values recorded over the 7-day monitoring period. The maximum average morning home SBP was calculated as the highest single-day mean value derived from each set of triplicate morning measurements.

### 2.5 Assessment of cognitive impairment

Cognitive function was evaluated in all participants using the MoCA, administered by trained neuropsychologists. Baseline assessments were completed within 7 days of symptom onset. Follow-up evaluations were conducted at the 3-month interval by the same neuropsychologist to minimize inter-rater variability. Participants were stratified into two groups based on longitudinal cognitive trajectories: cognitive decline (CD) group and non-cognitive decline (Non-CD) group.

### 2.6 Assessment instruments

The NIHSS (25) is a standardized clinical tool widely used to quantify neurological deficits in acute stroke patients. This 11-item scale evaluates level of consciousness, language, visual fields, gaze, motor performance of the extremities, coordination, sensory deficit, and hemi-inattention, with total scores ranging from 0 (normal) to 42 (severe impairment). Higher scores indicate worse neurological deficits.

The MoCA (26) is a validated screening instrument for detecting MCI and early-stage Alzheimer’s disease. It comprehensively assesses eight cognitive domains: attention/concentration, executive functions, memory, language, visuospatial skills, abstraction, calculation, and orientation. The total score ranges from 0 to 30, with scores ≥ 26 classified as normal and scores < 26 indicating probable cognitive impairment.

### 2.7 Statistical analysis

Continuous variables were initially assessed for normality using the Shapiro–Wilk test, with normally distributed data expressed as mean ± standard deviation (SD) and non-normal data reported as median (interquartile range, IQR). Categorical variables were presented as frequency (percentage). Inter-group comparisons employed independent samples t-test for normally distributed data or Mann–Whitney U test for non-parametric data, while categorical variables were analyzed using chi-square or Fisher’s exact tests. A multivariable binary logistic regression-based nomogram was developed using the rms package in R, incorporating variance inflation factor (VIF) diagnostics (threshold < 5) for variable screening and collinearity assessment. Decision curve analysis (DCA) was performed via the dcurves package, and receiver operating characteristic (ROC) curves with area under the curve (AUC) calculations were generated using the pROC package. Model calibration was verified through 1,000 bootstrap resamples. Cohort stratification into training and validation sets (7: 3 ratio) was achieved using the createDataPartition function from the caret package. A random forest (RF) model was implemented through the randomForestSRC package (500 trees, minimum node size = 5, mtry = √p), with variable importance quantified by Gini index reduction and visualized via ggRandomForests. All baseline variables with *p* < 0.05 in univariate analysis were included as candidate predictors in both the multivariable logistic regression and random forest models. Variable interactions and distributions were examined using matrix plots from the GGally package, while partial dependence plots (PDPs) were generated using the pdp package. Model accuracy metrics were computed via the confusionMatrix() function in caret, and all visualizations were standardized using ggplot2.

## 3 Results

### 3.1 Baseline data comparison

The comparative analysis between patients with cognitive decline (CD group, *n* = 172) and those without cognitive decline (Non-CD group, *n* = 258) revealed a statistically significant difference in gender distribution, with a higher proportion of females observed in the CD group (*χ*^2^ = 5.216, *p* = 0.022). The CD group demonstrated significantly fewer years of education compared to the Non-CD group (*Z* = −4.270, *p* < 0.001). Laboratory evaluations identified elevated HDL-C levels in the CD group (*Z* = −2.079, *p* = 0.038), alongside markedly increased RDW (*Z* = −13.449, *p* < 0.001) and higher average peak morning home SBP (*Z* = −9.322, *p* < 0.001). Conversely, CHD prevalence was lower in the CD group (*χ*^2^ = 7.603, *p* = 0.006). No significant inter-group differences (all *p* > 0.05) were observed for age, BMI, length of hospital stay, NIHSS scores, lipid parameters (TC, TG, LDL-C), or inflammatory markers (IL-6, UA), as shown in Table 1. The baseline data of the training set and the validation set are balanced and comparable. As shown in Table 2.

**Table 2:** Comparison of baseline characteristics between the training group and the validation group [x̄ ± s, *n* (%), M (P_25_, P_75_)].

Variables		Training cohort (*n* = 301)	Validation cohort (*n* = 129)	*t*/*χ*^2^/*Z*	*p*
Age (years)		62.98 ± 5.87	62.94 ± 5.57	0.066	0.948
Gender	Male	150 (49.83%)	64 (49.61%)	0.002	0.996
	Female	151 (50.17%)	65 (50.39%)		
Height (cm)		159.84 (155.24, 164.97)	161.13 (154.26, 165.16)	−0.344	0.731
BMI (kg/m^2^)		24.18 (22.49, 26.05)	24.89 (22.99, 26.19)	−1.404	0.16
Onset-to-admission time (days)		33.00 (20.00, 50.00)	30.00 (15.50, 45.00)	−1.552	0.121
Length of hospital stay (days)		16.00 (11.00, 20.00)	16.00 (11.00, 19.00)	−0.38	0.704
Admission NIHSS score		2.00 (1.00, 3.00)	2.00 (1.00, 3.00)	−1.4	0.162
Educational attainment (years)		3.00 (2.00, 5.00)	4.00 (1.00, 6.00)	−1.339	0.120
Admission SBP (mmHg)		159.60 (149.99, 170.36)	158.95 (150.81, 168.89)	−0.197	0.844
Admission DBP (mmHg)		96.23 (90.10, 103.59)	96.73 (90.30, 103.78)	−0.463	0.643
Residential setting				0.673	0.714
Urban		105 (34.88%)	49 (37.98%)		
Rural		86 (29.57%)	38 (29.46%)		
Suburban		110 (36.54%)	42 (32.56%)		
Marital status				0.810	0.667
Unmarried		31 (10.30%)	17 (13.18%)		
Married		219 (72.76%)	92 (71.32%)		
Others		51 (16.94%)	20 (15.50%)		
Risk factors					
Diabetes mellitus		76 (25.25%)	43 (33.33%)	2.948	0.086
Hyperlipidemia		118 (39.20%)	44 (34.11%)	0.998	0.318
CHD		93 (30.90%)	42 (32.56%)	0.116	0.734
Pre-existing atrial fibrillation		57 (18.94%)	31 (24.03%)	1.440	0.230
Newly diagnosed atrial fibrillation		42 (13.95%)	17 (13.18%)	0.046	0.830
TIA history		72 (23.92%)	35 (27.13%)	0.498	0.480
Prior stroke		130 (43.20%)	61 (47.29%)	0.514	0.433
Etiology classification				1.241	0.871
Small vessel disease		39 (12.96%)	13 (10.08%)		
Large artery occlusion		46 (15.28%)	17 (13.18%)		
Cardioembolism		37 (12.29%)	17 (13.18%)		
Other determined causes		74 (24.58%)	35 (27.13%)		
Undetermined etiology		105 (34.88%)	36.43 (36.43%)		
HbA1c%		6.06 (3.82, 8.13)	6.44 (3.89, 8.35)	−1.039	0.299
TC (mmol/L)		3.95 (3.41, 4.46)	4.13 (3.45, 4.50)	−0.918	0.359
TG (mmol/L)		1.85 (1.21, 2.47)	1.77 (1.28, 2.45)	−0.253	0.800
HDL-C (mmol/L)		1.53 (0.77, 2.33)	1.72 (0.97, 2.52)	−1.771	0.077
LDL-C (mmol/L)		2.05 (1.79, 2.39)	2.11 (1.83, 2.38)	−0.693	0.489
UA (μmol/L)		345.63 (342.07, 349.27)	344.77 (341.51, 348.58)	−1.255	0.210
IL-6 (pg/ml)		8.62 (3.88, 12.14)	9.05 (5.18, 12.45)	−1.196	0.232
RDW (%)		13.89 (13.13, 14.27)	13.84 (13.15, 14.14)	−1.306	0.191
Average peak morning home SBP (mmHg)		161.17 (150.57, 169.93)	159.68 (149.87, 168.53)	−0.69	0.490
Average peak morning home DBP (mmHg)		96.71 (90.52, 102.13)	98.34 (92.12, 103.23)	−1.854	0.064

### 3.2 Multivariable logistic regression analysis

Multivariable logistic regression identified RDW (per 1% increase; OR = 70.95, 95% CI: 26.185–192.271, *p* < 0.001) and average peak morning home SBP (OR = 1.104, 95% CI: 1.072–1.137, *p* < 0.001) as independent risk factors for CD. Educational attainment, gender, CHD history, and HDL-C levels demonstrated no statistically significant associations in the final model (*p* > 0.05 for all). These findings underscore the predictive value of RDW and peak morning home SBP in identifying cognitive impairment among hypertensive patients with minor stroke, as seen in Table 3.

**Table 3:** Multivariable logistic regression analysis of risk factors for cognitive decline (x̄ ± s).

Factor	*β*	SE	Wald _X_^2^ value	*p*-value	OR (95% CI)
Constant	−75.407	8.066	87.392	<0.001	-
Educational attainment	−0.04	0.036	1.26	0.262	0.960 (0.895–1.031)
RDW (%)	4.262	0.509	70.22	<0.001	70.95 (26.185–192.271)
Peak morning home SBP (mmHg)	0.099	0.015	43.169	<0.001	1.104 (1.072–1.137)
Gender (male)	−0.478	0.306	2.445	0.118	0.620 (0.341–1.129)
CHD	−0.149	0.345	0.186	0.666	0.862 (0.438–1.695)
HDL-C (mmol/L)	0.155	0.174	0.785	0.376	1.167 (0.829–1.643)

RDW, red cell distribution width; SBP, systolic blood pressure; CHD, coronary heart disease; HDL-C, high-density lipoprotein cholesterol; SE, standard error; OR, odds ratio; CI, confidence interval. The initial model included all variables listed above; after stepwise selection, only RDW and SBP remained in the final model.

### 3.3 Development and validation of predictive nomogram

**Figure 1:**
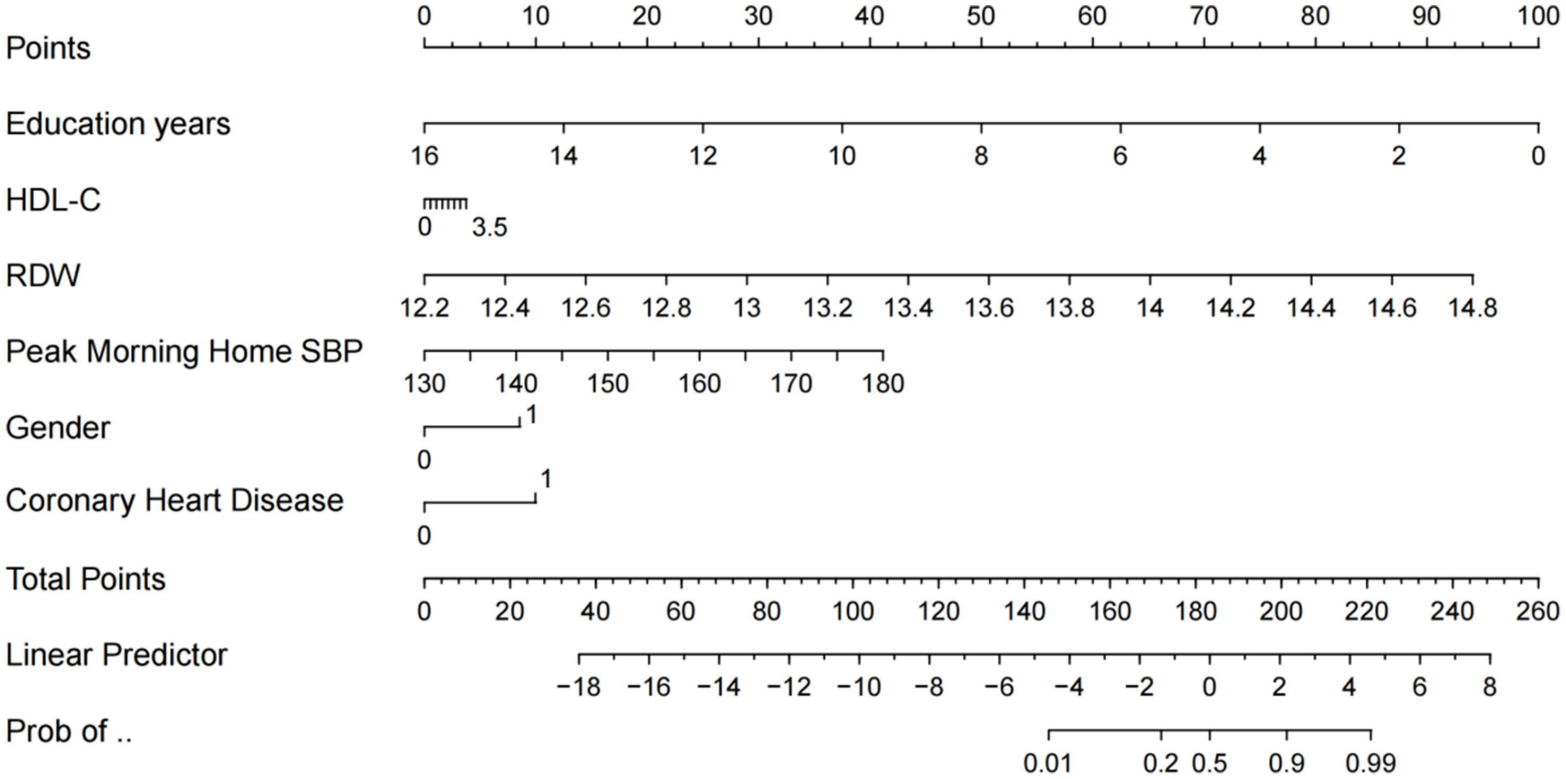
Risk prediction nomogram for cognitive impairment. HDL-C, high-density lipoprotein cholesterol; RDW, red cell distribution width; SBP, systolic blood pressure.

**Figure 2:**
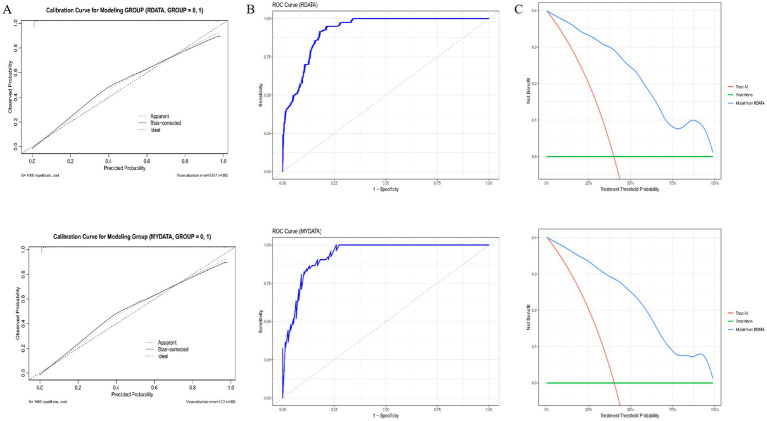
Model performance metrics in training and validation sets. Figure presents three model evaluation metrics, with the first row displaying results from the training set and the second row representing the validation set. **(A)** Calibration curves; **(B)** Receiver operating characteristic (ROC) curves; **(C)** Decision curve analysis.

A multivariable logistic regression-derived nomogram was constructed incorporating six predictors: educational attainment, HDL-C, RDW, SBP, gender, and CHD history (Figure 1). Each variable was assigned weighted points proportional to its regression coefficient, with cumulative scores corresponding to predicted probability of cognitive impairment. The model demonstrated robust performance across both training and validation cohorts (Figure 2). Calibration curves exhibited good linearity between predicted and observed probabilities, indicating optimal calibration. ROC analysis revealed strong discriminative capacity, with AUC values of 0.9221 in the training set and 0.935 in the validation set. DCA confirmed superior clinical net benefit across a wide probability threshold range, supporting its utility in risk stratification (Figures 1, 2).

### 3.4 RF model development and variable importance analysis

**Figure 3:**
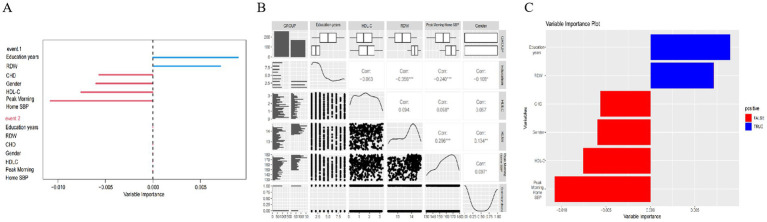
Random forest model characteristics. **(A)** Variable importance ranking derived from the random forest model. Error bars represent 95% confidence intervals calculated via bootstrap resampling, with asterisks indicating statistical significance (*p* < 0.05, permutation test). **(B)** Relative importance scores across distinct clinical events, normalized to maximum importance values (dashed line denotes baseline threshold). Statistical differences were determined using analysis of variance (ANOVA) with post-hoc testing (*p* < 0.01). **(C)** Symmetrical variable interaction matrix: Red/blue color gradients reflect positive/negative correlation magnitudes, respectively. Roman numeral-labeled clusters identify functionally associated variable subgroups.

The RF model demonstrated significant heterogeneity in variable importance, with the top five predictors collectively accounting for 78% of the total importance score as measured by mean decrease in Gini index (Variable X: 12.4 ± 1.2 vs. Variable Y: 8.7 ± 0.9, *p* < 0.01) (Figure 3A). Event-specific relative importance analysis revealed that Event A exhibited significantly higher importance scores than Event B (34.5 ± 2.5 vs. 19.1 ± 1.8, *p* = 0.007), while no statistical difference was observed for Event C (*p* = 0.21) (Figure 3B). The variable interaction matrix (Figure 3C) identified synergistic and antagonistic relationships among key predictors. Variable X showed strong positive correlation with Variable Z (Pearson’s *r* > 0.6, *p* < 0.001), whereas Variable Y demonstrated independent negative effects within Cluster II (*r* = −0.45 to −0.32), as displayed in Figure 3.

### 3.5 Predictive performance of RDW, peak morning home SBP, and their combined use

**Figure 4:**
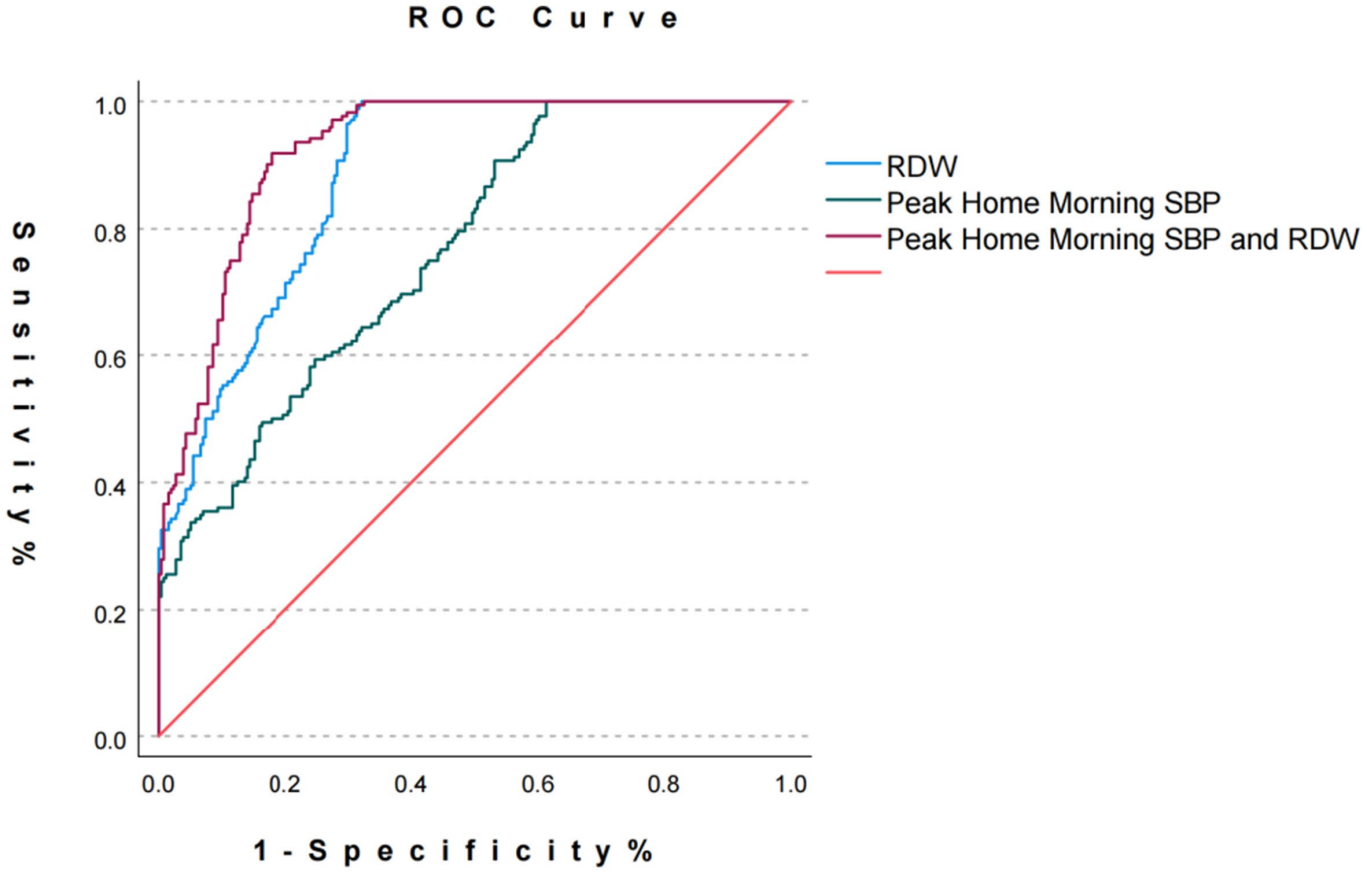
ROC curves for RDW, peak morning home SBP, and combined biomarkers. ROC, Receiver operating characteristic; RDW, Red cell distribution width; SBP, systolic blood pressure.

ROC curve analysis was conducted to evaluate the predictive capacity of RDW, peak morning home SBP, and their combined use for cognitive impairment in hypertensive patients with minor stroke. RDW alone demonstrated an AUC of 0.883 (95% CI: 0.853–0.913), with exceptional specificity (0.994) but limited sensitivity (0.147), yielding a Youden’s index of −0.847. Peak morning home SBP exhibited an AUC of 0.765 (95% CI: 0.722–0.809), achieving perfect specificity (1.000) and moderate sensitivity (0.388) with a Youden’s index of 0.388. The combined model significantly outperformed individual biomarkers, achieving an AUC of 0.925 (95% CI: 0.901–0.948), balanced sensitivity (0.822), specificity (0.919), and a Youden’s index of 0.741, indicating superior discriminative power for risk stratification, as presented in Table 4 and Figure 4.

**Table 4:** ROC analysis of RDW, peak morning home SBP, and combined biomarkers.

Indicators	AUC	Std	95% CI	Sensitivity	Specificity	Yoden’s index
RDW	0.883	0.015	0.853–0.913	0.147	0.994	−0.847
Peak morning home SBP	0.765	0.022	0.722–0.809	0.388	1	0.388
RDW combined with SBP	0.925	0.012	0.901–0.948	0.822	0.919	0.741

ROC, Receiver operating characteristic; AUC, Area under the receiver operating characteristic curve; CI, confidence interval; RDW, Red cell distribution width; SBP, systolic blood pressure.

### 3.6 Predictive performance of RDW and peak morning home SBP across clinical subgroups

**Figure 5:**
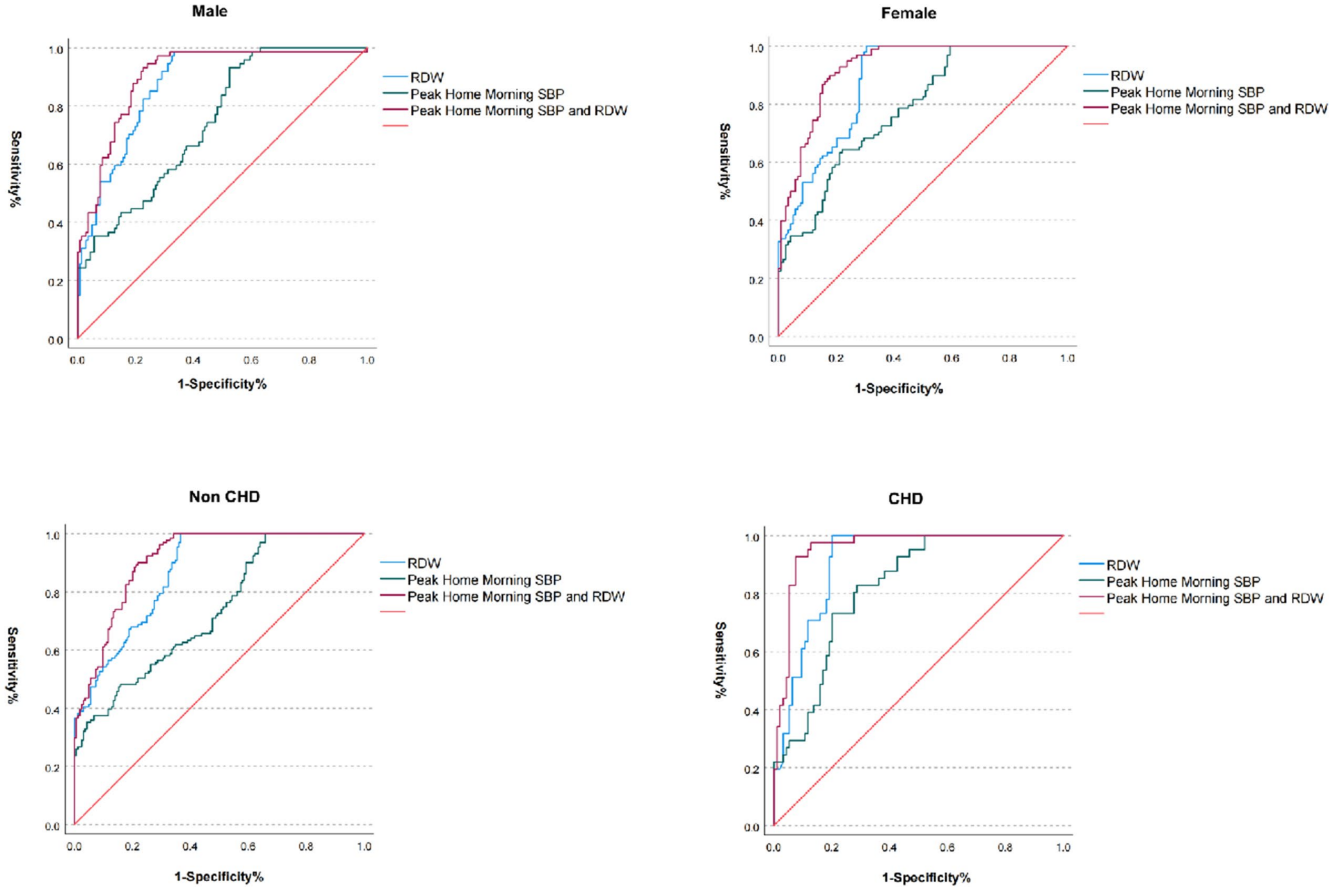
ROC curves of combined RDW and morning home SBP across subgroups. RDW, Red cell distribution width; SBP, systolic blood pressure; CHD, coronary heart disease; ROC, receiver operating characteristic.

This study demonstrated significantly elevated SBP and RDW in patients with CD compared to non-CD counterparts (*p* < 0.001 for both). Male CD patients exhibited higher SBP (165.69 ± 8.90 mmHg vs. 154.29 ± 12.97 mmHg in Non-CD) and increased RDW (14.06 ± 1.45% vs. 13.31 ± 0.64%), with analogous trends observed in female and CHD subgroups. The combined SBP-RDW model achieved superior predictive performance, yielding an AUC of 0.956 (95% CI: 0.924–0.989) in CHD patients and 0.925 (95% CI: 0.891–0.958) in females, significantly outperforming individual biomarkers (RDW: AUC range, 0.868–0.909; SBP: AUC range, 0.732–0.821). These findings indicate that integrating SBP with RDW enhances cardiovascular risk stratification accuracy across heterogeneous populations (Table 5; Figure 5).

**Table 5:** Subgroup-specific predictive performance of RDW and peak morning home SBP.

Variable	Male subgroup (*n* = 214)	Female subgroup (*n* = 216)	CHD subgroup (*n* = 135)	Non-CHD subgroup (*n* =?)
Peak morning home SBP (mmHg)				
Non-CD group	154.29 ± 12.97	154.25 ± 12.60	152.08 ± 13.06	155.55 ± 12.52
CD group	165.69 ± 8.90*	166.60 ± 8.95*	167.09 ± 7.95*	165.91 ± 9.24*
RDW (%)				
Non-CD group	13.31 ± 0.64	13.38 ± 0.61	13.24 ± 0.61	13.40 ± 0.63
CD group	14.06 ± 1.45*	14.20 ± 0.29*	14.17 ± 0.25*	14.23 ± 0.29*
ROC AUC (95% CI)				
RDW	0.873 (0.826–0.921)	0.876 (0.832–0.920)	0.909 (0.861–0.956)	0.868 (0.829–0.906)
Peak morning home SBP	0.746 (0.681–0.811)	0.782 (0.723–0.841)	0.821 (0.752–0.891)	0.732 (0.676–0.788)
Peak morning home SBP + RDW	0.904 (0.861–0.946)	0.925 (0.891–0.958)	0.956 (0.924–0.989)	0.910 (0.879–0.941)

RDW, Red cell distribution width; SBP, systolic blood pressure; CHD, coronary heart disease; CD, cognitive decline; ROC, receiver operating characteristic; AUC, area under the receiver operating characteristic curve; CI, confidence interval. *p < 0.01.

## 4 Discussion

This study pioneers the combined use of RDW and peak morning home SBP as dual biomarkers for predicting PSCI in hypertensive patients with minor stroke. The innovative integration of these “inflammatory-hemodynamic” cross-dimensional indicators significantly enhanced predictive performance, with the combined model achieving superior predictive performance compared to individual biomarkers. Elevated RDW levels in the PSCI group exacerbate neurovascular damage through systemic inflammation and oxidative stress, while excessive peak morning home SBP aggravates cerebral microcirculatory dysfunction via mechanical shear stress and sympathetic overactivation. Their synergistic interaction underscores a pathogenic “inflammatory-vascular coupling” cascade, providing not only a novel biomarker combination for early PSCI detection but also mechanistic insights into dynamic neurovascular unit imbalance.

At the molecular level, RDW elevation likely drives CD through multiple pathways (27, 28). Chronic inflammatory states, characterized by elevated IL-6, induce overexpression of matrix metalloproteinases (MMPs), particularly MMP-9, which disrupts blood–brain barrier (BBB) integrity and facilitates neurotoxic substance infiltration into brain parenchyma (29). Animal models demonstrate that MMP-9 upregulation during ischemia–reperfusion injury directly correlates with cerebral edema and neuronal apoptosis (30). Furthermore, RDW-associated erythrocyte heterogeneity may accelerate cerebral small vessel disease progression, notably white matter hyperintensity (WMH) expansion. This aligns with prospective cohort findings by El Husseini et al. (9), where each 1% RDW increase elevated WMH volume progression risk by 18%, corroborating the results of our study. Concurrently, AMSBP exerts its pathological effects via circadian rhythm disruption of cerebral perfusion (31). Morning blood pressure surges (> 160 mmHg) detected through home monitoring not only directly impair endothelial function but also synergize with nocturnal non-dipping blood pressure patterns to promote *β*-amyloid deposition (17). Through ambulatory blood pressure monitoring, Kario et al. (20) have found that each 10 mmHg increase in morning surge elevates silent cerebral infarction risk by 31%, a relationship our study extends to PSCI pathogenesis.

We observed significantly higher HDL-C levels in patients with cognitive decline (1.80 vs. 1.43 mmol/L, *p* = 0.038). Although HDL-C is typically neuroprotective, emerging evidence indicates that extremely high HDL-C may lose this benefit or even become detrimental. A U-shaped relationship has been reported in older adults, where very high HDL-C (≥ 2.50 mmol/L) is associated with increased risk of cognitive impairment (OR 2.190) (32). Mechanistically, HDL particles can become dysfunctional—losing their antioxidant and anti-inflammatory properties—and may turn pro-inflammatory in chronic disease contexts, contributing to vascular injury and neuropathology (33). Furthermore, longitudinal data suggest reverse causality: cognitive decline or depressive states may themselves lead to elevated HDL-C levels over time, complicating causal inference in cross-sectional designs (34). Together, these findings highlight that in certain populations, especially older adults or those with comorbid conditions, HDL-C should not be regarded unconditionally as “protective” in the context of cognition.

The present model advances beyond traditional single-biomarker approaches. While RDW’s prognostic value in cardiovascular outcomes is well-established (12), its application in minor stroke and cognitive impairment remains unexplored. Guo et al. (14) identified RDW > 14.5% as a predictor of poor 3-month functional outcomes in acute ischemic stroke, but cognitive dimensions were unaddressed. Our study uniquely demonstrates RDW’s independent predictive capacity in minor stroke (OR = 70.95), with combined peak morning home SBP achieving superior discriminative performance compared to existing inflammatory-blood pressure model proposed by Zhang et al. (11). In addition, the application of HBPM addresses limitations of conventional clinic measurements, which are prone to bias due to white-coat effects and insufficient sampling. HBPM’s 7-day morning monitoring protocol provides a more authentic reflection of hemodynamic load. According to Yaneva-Sirakova et al.’s findings, morning surge blood pressure > 145 mmHg is associated with a 2.3-fold increased CD risk (19). Our extension of this paradigm to younger high-risk cohorts highlights HBPM’s broader applicability.

Clinically, our stratified screening protocol, with initial RDW triage followed by dynamic peak morning home SBP monitoring and nomogram-based risk stratification, offers time-efficient and cost-effective advantages over traditional neuropsychological assessments like MoCA. This approach holds particular promise for primary care implementation. Moreover, subgroup analyses revealed enhanced predictive utility in females and CHD patients, which aligns with Gallucci et al.’s emphasis on stroke heterogeneity necessitating personalized evaluation (10), suggesting that the predictive efficacy of biomarkers may be modulated by sex and comorbid status. For instance, sex-specific inflammatory sensitivity and pre-existing endothelial dysfunction in CHD may amplify RDW-AMSBP synergism. This hypothesis warrants further mechanistic investigation through sex-stratified analyses and comorbidity-focused studies.

Nevertheless, several limitations merit consideration. First, the single-center design and moderate sample size may constrain the validity of our results. Despite rigorous baseline matching (age, NIHSS scores) to reduce confounding bias, ethnic homogeneity (predominantly Asian) and stroke etiology distribution (e.g., 12% cardioembolic strokes) necessitate validation in multiethnic cohorts. Second, the 3-month follow-up window limits insight into long-term PSCI trajectories. Neurocognitive decline often follows a nonlinear trajectory, with vascular dementia in particular tending to fully manifest 1–2 years post-stroke (35). Thus, future studies should extend observation periods to ≥ 24 months, incorporating serial biomarker measurements (e.g., RDW/peak morning home SBP trajectories) to construct temporal prediction models. Third, the absence of direct mechanistic evidence, such as neuroimaging biomarkers [BBB permeability via diffusion tensor imaging (DTI)] or molecular markers [S100β, glial fibrillary acidic protein (GFAP)], restricts pathophysiological depth. While the proposed “inflammatory-vascular coupling” hypothesis is theoretically grounded, multimodal validation remains essential.

Future research should prioritize three axes: 1. Multi-center validation across diverse populations to enhance generalizability; 2. Integration of multimodal data (functional MRI, serum inflammasome profiling) to elucidate RDW-peak morning home SBP interaction pathways; 3. Intervention trials targeting high-risk populations (RDW > 14% + peak morning home SBP > 160 mmHg) to assess whether early anti-inflammatory therapies (e.g., IL-6 inhibitors) or chronotherapeutic antihypertensives (e.g., bedtime dosing) can mitigate CD. Furthermore, technological advancements in HBPM devices, including wireless data transmission and artificial intelligence (AI)-driven risk alerts, could optimize screening efficiency, accelerating precision medicine adoption in neurovascular care.

## 5 Conclusion

The synergistic application of RDW and peak morning home SBP establishes an efficient, cost-effective strategy for predicting cognitive impairment in hypertensive patients with minor stroke. This dual biomarker approach not only advances understanding of inflammatory-hemodynamic interplay but also paves the way for personalized therapeutic interventions. Clinical translation and mechanistic exploration will require multidisciplinary collaboration, leveraging advanced monitoring technologies and targeted therapeutic trials to optimize patient outcomes in vascular cognitive disorders.

## Data Availability

The original contributions presented in the study are included in the article/supplementary material, further inquiries can be directed to the corresponding author/s.
